# Study on the non-linear relationship between neutrophil-to-lymphocyte ratio and prognosis of spontaneous intraventricular hemorrhage

**DOI:** 10.3389/fneur.2025.1768967

**Published:** 2026-01-15

**Authors:** Jun-Jian Zhou, Ming Lu, Wen-Bin Dai

**Affiliations:** 1 The Second School of Clinical Medicine, Zhejiang Chinese Medical University, HangZhou, China; 2 Department of Neurosurgery, The First People's Hospital of Xiaoshan District of Hangzhou City, HangZhou, China

**Keywords:** inflammatory biomarker, neutrophil-to-lymphocyte ratio, nonlinear relationship, prognosis, spontaneous intraventricular hemorrhage

## Abstract

**Background:**

The neutrophil-to-lymphocyte ratio (NLR), as an inflammatory marker, has been shown to be associated with the prognosis of various cerebrovascular diseases. However, the specific non-linear relationship between NLR and the prognosis of spontaneous intraventricular hemorrhage (sIVH) remains unclear. This study aimed to investigate the non-linear association between NLR and poor outcomes in sIVH patients.

**Methods:**

This retrospective study analyzed data from Xiaoshan First People’s Hospital, including all patients hospitalized for sIVH. Blood samples were collected immediately upon hospital admission to calculate NLR, and its relationship with 90-day functional outcomes (defined as poor prognosis with a modified Rankin Scale score ≥4) was evaluated.

**Results:**

The analysis revealed a J-shaped relationship between NLR and poor outcomes in sIVH patients. As NLR levels increased, the risk of poor outcomes rose, reaching saturation at approximately 8.26. Specifically, when NLR was below 8.26, the odds ratio was 1.49 (95% CI: 1.16–1.91, *p* = 0.0018); when NLR was above 8.26, the odds ratio was 0.95 (95% CI: 0.84–1.07, *p* = 0.4194). Multivariate analysis indicated that NLR was an independent predictor of 90-day prognosis in sIVH patients.

**Conclusion:**

NLR can serve as an important indicator for assessing the prognosis of sIVH patients. The non-linear relationship between NLR and poor outcomes provides new insights for clinical management. Further studies should explore the mechanisms of NLR and its potential applications in sIVH treatment.

## 1 Introduction

Based on the provided references, the following synthesis can be made regarding the role of the neutrophil-to-lymphocyte ratio (NLR) as a prognostic biomarker in spontaneous intraventricular hemorrhage (sIVH): Spontaneous intraventricular hemorrhage (sIVH) is a severe form of stroke with high morbidity and mortality rates (1). Despite advancements in medical technologies, the prognosis for patients with sIVH remains poor, and therapeutic interventions are limited (2). This underscores the urgent need for reliable prognostic biomarkers that can facilitate early risk stratification and potentially guide targeted interventions (3).

Inflammation is a critical factor in the pathogenesis and progression of cerebrovascular diseases, including sIVH (4). The neutrophil-to-lymphocyte ratio (NLR), a simple and readily available inflammatory marker, has emerged as a promising prognostic indicator across various neurological disorders (5). Studies have shown that elevated NLR is associated with poorer outcomes in conditions such as ischemic stroke, traumatic brain injury, and intracerebral hemorrhage (5–7).

While several studies have demonstrated significant associations between elevated neutrophil-to-lymphocyte ratio (NLR) and adverse neurological outcomes in acute ischemic stroke patients, including early neurological deterioration and stroke-associated complications (8, 9), evidence regarding the relationship between NLR and prognosis in hemorrhagic stroke remains limited. More importantly, no studies have specifically investigated the potential non-linear (e.g., J-shaped or U-shaped) relationship between NLR and clinical outcomes in spontaneous intraventricular hemorrhage (sIVH) patients. This knowledge gap represents a significant limitation in our understanding of inflammatory biomarkers in sIVH prognosis and forms the primary focus of the present study. To our knowledge, no studies have specifically investigated this non-linear relationship in spontaneous intraventricular hemorrhage (sIVH) populations, indicating a gap in the current understanding of its utility in this context (3). Most existing prognostic models for intracerebral hemorrhage primarily focus on initial hemorrhage volume, location, and early neurological deterioration (10). These traditional approaches often overlook the complex inflammatory cascades that significantly influence patient outcomes (3).

The potential of inflammatory biomarkers like NLR to provide additional prognostic information has not been fully investigated in sIVH patients, suggesting that integrating such biomarkers into existing models could enhance predictive accuracy and improve patient management strategies (3, 11). Furthermore, the timing of measuring inflammatory markers may be crucial, as interventions administered within 24 h of stroke have shown the largest effect sizes (12).

In conclusion, the exploration of inflammatory markers such as NLR in the context of sIVH could provide valuable insights into patient prognosis and inform therapeutic decisions (3). Given the established role of inflammation in cerebrovascular diseases, further research is warranted to elucidate the specific mechanisms by which NLR and other inflammatory markers influence outcomes in sIVH (3, 4, 7).

## 2 Materials and methods

### 2.1 Research participant

A single-center retrospective study was conducted at our institution. Data were collected from patients admitted with spontaneous intraventricular hemorrhage and who underwent external ventricular drainage (EVD) between 2016 and 2024. The specific inclusion and exclusion criteria are as follows:

Inclusion criteria: a. Age 18–80 years; b. First onset, with cranial CT examination confirming the diagnosis of spontaneous intraventricular hemorrhage; c. Patients have complete clinical data, including imaging examination results, biochemical indicators, and medical history; d. Regular and complete follow-up.

**Figure 1:**
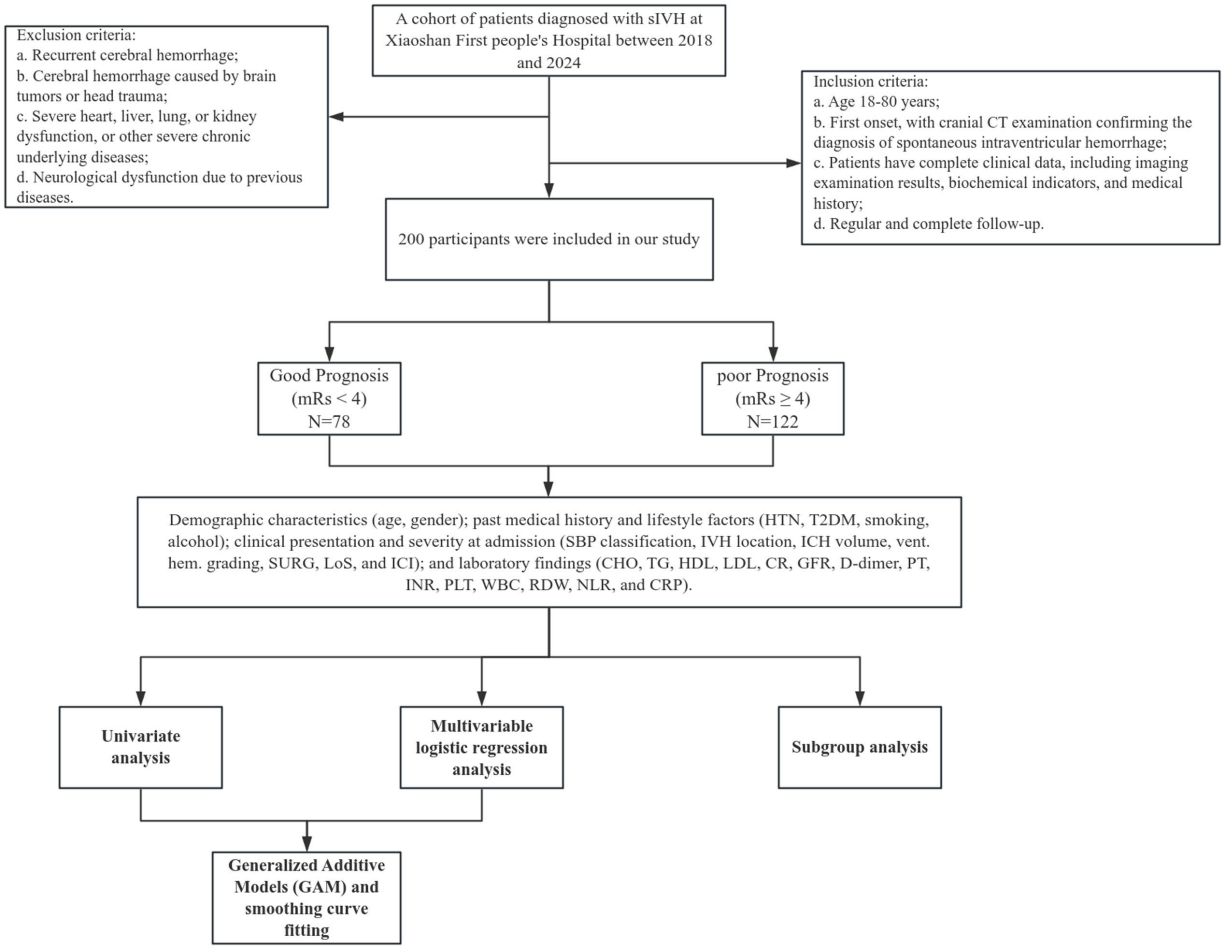
Flowchart of the study participants.

Exclusion criteria: a. Recurrent cerebral hemorrhage; b. Cerebral hemorrhage caused by brain tumors or head trauma; c. Severe heart, liver, lung, or kidney dysfunction, or other severe chronic underlying diseases; d. Neurological dysfunction due to previous diseases (Figure 1).

### 2.2 Definition of spontaneous intraventricular hemorrhage

Spontaneous intraventricular hemorrhage (sIVH) is a serious medical condition characterized by the rupture of intracranial blood vessels and the subsequent entry of blood into the ventricular system (13). It is estimated to account for approximately 20–60% of intracerebral hemorrhage (ICH) cases (13). sIVH can be classified into primary and secondary types based on the location of the hemorrhage (13). Primary IVH refers to bleeding occurring within 1.5 cm of the ventricular choroid plexus or subcommissural area, while secondary IVH results from bleeding in other areas, such as the cerebral lobes, thalamus, or basal ganglia, which subsequently spills into the ventricles (13). Approximately 93% of IVH cases are secondary in nature (13). The mortality rate associated with sIVH is estimated to range from 23 to 83%, which is nearly three times higher than that observed in patients without IVH (13). This high mortality rate is likely due to the severe complications associated with sIVH, such as increased intracranial pressure, hydrocephalus, and brain herniation (13). Several factors have been identified as potential causes of sIVH, including anticoagulation therapy (14), meningiomas (15–17), arteriovenous malformations (18), and venous sinus thrombosis (19). Additionally, some studies have suggested that certain medical conditions, such as Guillain-Barré syndrome (20, 21), may be associated with the development of sIVH. The management of sIVH often involves a multifaceted approach, including neurocritical care, maintenance of adequate cerebral perfusion pressure, and the use of extraventricular drainage (EVD) to manage hydrocephalus (13). Some studies have also investigated the use of intraventricular fibrinolysis (IVF) as a potential treatment option, but the results have been mixed (22).

### 2.3 Laboratory data collection

Complete blood count analysis was performed on venous blood samples collected immediately upon hospital admission for all patients as part of standard emergency department evaluation protocols. The neutrophil-to-lymphocyte ratio (NLR) was calculated by dividing the absolute neutrophil count by the absolute lymphocyte count from the admission blood sample.

### 2.4 Covariates

In accordance with the findings of previous research, the following data were collected prior to the commencement of treatment: The data set included Graeb scores, imaging features (location of primary hemorrhage, location of intraventricular hemorrhage, hydrocephalus, volume of fourth ventricle hemorrhage, volume of parenchymal hemorrhage), and relevant clinical factors (general condition: age, sex, SBP, DBP, Smoking and drinking habits); Comorbidities: history of hypertension, atrial fibrillation, coronary artery disease, type 2 diabetes, long-term anticoagulation treatment, moyamoya disease, aneurysm, arteriovenous malformation; Complications: intracranial infection; Biochemical indicators: The following biochemical indicators were also recorded: cholesterol (CHO), triglycerides (TG), high-density lipoprotein (HDL), low-density lipoprotein (LDL), platelets (PLT), international normalized ratio (INR), neutrophils (NEU), blood glucose (BG), prothrombin time (PT), glomerular filtration rate (GFR), D-dimer, creatinine, and C-reactive protein. Additionally, the following factors were documented: duration of drainage tube placement, use of urokinase, treatment group, tracheotomy, duration of intensive care unit (ICU) stay, and other indicators.

### 2.5 Treatment overview

All patients received standard institutional care for spontaneous intraventricular hemorrhage. Treatment was individualized based on clinical presentation, Glasgow Coma Scale score, imaging findings, and patient condition. Some patients (*n* = 171, 85.5%) underwent external ventricular drainage (EVD) for hydrocephalus or elevated intracranial pressure, while others (*n* = 29, 14.5%) received conservative management including blood pressure control, intracranial pressure monitoring, and supportive care. This study primarily evaluated the prognostic value of neutrophil-to-lymphocyte ratio in the overall sIVH population, regardless of treatment modality. Treatment approach was recorded as a potential confounder but not the primary variable of interest.

### 2.6 Postoperative patient management

In the EVD group, we gave 5 mL of saline mixed with 20–30,000 units of urokinase every 4–6 h postoperatively in order to liquefy the hematoma. Regular cranial CT examinations were performed to assess the effect of hematoma removal, and the drainage tube was removed when the hematoma disappeared or the remaining hematoma volume was less than 10 mL and the patient’s vital signs were stable. Peripheral blood specimens were collected daily at 7:00 a.m. after surgery. Patient prognosis was assessed using the modified Rankin Scale (mRS) to evaluate survival and functional outcomes at 90 days, but it is important to note that multiple patients remained hospitalized for over 90 days, which reflects the severity of their clinical conditions. Patients with mRs ≥ 4 defined as poor prognosis and mRs < 4 as good prognosis at 90 days postoperatively.

### 2.7 Statistical analysis

Data are presented as mean ± SD, median (IQR), or frequency (%). Group comparisons utilized Student’s t-test, Mann–Whitney U test, Chi-square test, or Fisher’s exact test, as appropriate. Multivariate logistic regression was performed to determine the independent association between NLR and functional outcomes across three hierarchical models. We verified the absence of multicollinearity among covariates using the Variance Inflation Factor (VIF), with a threshold of < 5 indicating no significant collinearity. To characterize non-linear relationships, we employed generalized additive models (GAM) with smooth curve fitting. Threshold effects were calculated using two-piecewise linear regression, and likelihood ratio tests were used to determine statistical significance. Subgroup analyses were conducted to examine potential interactions. All analyses were performed using R software^1^ and EmpowerStats (X&Y Solutions, Inc., Boston, MA), with statistical significance set at two-sided *p* < 0.05.

## 3 Results

### 3.1 Baseline characteristics

Table 1 summarizes the clinical and laboratory characteristics of patients with secondary intraventricular hemorrhage (sIVH) based on prognosis. The analysis indicates that patients with a Poor prognosis exhibit significantly larger intracerebral hemorrhage volumes (*p* = 0.001) and higher inflammatory markers, including neutrophil-to-lymphocyte ratio (NLR, *p* < 0.001) and white blood cell counts (*p* = 0.008), suggesting a close association between systemic inflammation and adverse outcomes. Additionally, these patients showed increased D-dimer levels (*p* = 0.012) and a higher prevalence of hypertension (*p* = 0.053), indicating potential coagulation issues and comorbid conditions. Notably, the Poor prognosis group required surgical intervention more frequently (*p* < 0.001) and had a greater incidence of intracranial infections (*p* = 0.016).

**Table 1:** Weighted characteristics of the study population based on sIVH.

Characteristic	Good prognosis (*n* = 78)	Poor prognosis (*n* = 122)	*p*-value
CHO (Mean ± SD)	4.44 ± 2.89	4.02 ± 1.24	0.146
TG (Mean ± SD)	1.81 ± 1.88	2.29 ± 3.23	0.167
HDL (Mean ± SD)	1.24 ± 0.27	1.24 ± 0.28	0.817
LDL (Mean ± SD)	2.23 ± 0.62	2.28 ± 1.08	0.428
GFR (Mean ± SD)	104.47 ± 26.92	96.07 ± 37.60	0.245
CR (Mean ± SD)	65.98 ± 16.95	95.18 ± 117.56	0.118
D. DIMER (Mean ± SD)	1.60 ± 4.01	1.89 ± 2.60	0.012
PT (Mean ± SD)	11.18 ± 0.91	11.95 ± 3.62	0.059
INR (Mean ± SD)	1.24 ± 1.61	1.04 ± 0.33	0.400
PLT (Mean ± SD)	206.92 ± 54.67	194.35 ± 65.12	0.134
ICH. VOL (Mean ± SD)	7.38 ± 9.53	14.68 ± 15.77	0.001
WBC (Mean ± SD)	10.87 ± 3.80	12.94 ± 5.12	0.008
RDW (Mean ± SD)	12.86 ± 1.36	12.95 ± 1.36	0.596
NLR (Mean ± SD)	11.03 ± 9.06	14.98 ± 9.36	<0.001
LoS (Mean ± SD)	23.97 ± 12.01	21.61 ± 15.61	0.132
AGE(years)			0.148
≤40	3 (3.85%)	10 (8.20%)	
>40, ≤60	44 (56.41%)	53 (43.44%)	
>60	31 (39.74%)	59 (48.36%)	
GENDER (%)			0.403
Male	42 (53.85%)	73 (59.84%)	
Female	36 (46. 15%)	49 (40. 16%)	
SBP (%)			0.417
≤140	29 (37. 18%)	40 (32.79%)	
>140, ≤160	23 (29.49%)	30 (24.59%)	
>160	26 (33.33%)	52 (42.62%)	
SMOKING (%)			0.149
No	49 (62.82%)	64 (52.46%)	
Yes	29 (37. 18%)	58 (47.54%)	
ALCOHOL (%)			0.142
No	51 (65.38%)	67 (54.92%)	
Yes	27 (34.62%)	55 (45.08%)	
HTN (%)			0.053
No	24 (30.77%)	23 (18.85%)	
Yes	54 (69.23%)	99 (81. 15%)	
T2DM (%)			0.402
No	68 (87. 18%)	101 (82.79%)	
Yes	10 (12.82%)	21 (17.21%)	
NLR (%)			0.017
≤ 10	44 (56.41%)	44 (36.07%)	
>10, ≤20	25 (32.05%)	54 (44.26%)	
>20	9 (11.54%)	24 (19.67%)	
CRP (%)			0.266
≤50	74 (96. 10%)	108 (89.26%)	
>50, ≤100	2 (2.60%)	8 (6.61%)	
>100	1 (1.30%)	5 (4. 13%)	
IVH. LOCATION (%)			0.009
IV, CL, SAS	16 (20.51%)	8 (6.56%)	
BG, Th	11 (14. 10%)	15 (12.30%)	
Cb, BS, MH	51 (65.38%)	99 (81. 15%)	
VENT. HEM (%)			0.063
1	16 (20.51%)	11 (9.02%)	
2	12 (15.38%)	13 (10.66%)	
3	15 (19.23%)	27 (22. 13%)	
4	35 (44.87%)	71 (58.20%)	
SURG (%)			<0.001
No	21 (26.92%)	8 (6.56%)	
Yes	57 (73.08%)	114 (93.44%)	
ICI (%)		0.016	0.016
No	70 (89.74%)	93 (76.23%)	
Yes	8 (10.26%)	29 (23.77%)	

CHO, Cholesterol; TG, Triglycerides; HDL, High-Density Lipoprotein; LDL, Low-Density Lipoprotein; GFR, Glomerular Filtration Rate; CR, Creatinine; D. DIMER, D-Dimer; PT, Prothrombin Time; INR, International Normalized Ratio; PLT, Platelet Count; ICH. VOL, Intracerebral Hemorrhage Volume; WBC, White Blood Cell Count; RDW, Red Cell Distribution Width; NLR, Neutrophil-to-Lymphocyte Ratio; SBP, Systolic Blood Pressure; HTN, Hypertension; T2DM, Type 2 Diabetes Mellitus; CRP, C-Reactive Protein; IVH. LOCATION, Location of Intraventricular Hemorrhage; VENT. HEM, Ventricular Hemorrhage; SURG, Surgery; ICI, Intracranial Infection.

### 3.2 Univariate analyses

Table 2 presents a univariate analysis of various clinical parameters and their association with prognosis in patients with secondary intraventricular hemorrhage (sIVH). Notably, the neutrophil-to-lymphocyte ratio (NLR) is a significant predictor of adverse outcomes, with an odds ratio (OR) of 1.05 (95% CI: 1.02, 1.09, *p* = 0.0051), indicating that each unit increase in NLR correlates with a 5% increase in the odds of a negative prognosis. The analysis also highlights that obstructive hydrocephalus severity significantly impacts prognosis, with mild and severe cases having ORs of 2.71 (*p* = 0.0204) and 4.67 (*p* < 0.0001), respectively. Additionally, midline shift greater than 15 mm is associated with an OR of 9.25 (*p* = 0.0345), signifying a critical prognostic factor.

**Table 2:** The association between NLR and sIVH.

Outcome	Statistics	PROG
AGE		
≤40	13 (6.50%)	1.0
>40, ≤60	97 (48.50%)	0.36 (0.09, 1.39) 0.1397
>60	90 (45.00%)	0.57 (0.15, 2.23) 0.4198
SBP		
≤140	69 (34.50%)	1.0
>140, ≤160	53 (26.50%)	0.95 (0.46, 1.95) 0.8797
>160	78 (39.00%)	1.45 (0.74, 2.84) 0.2777
ICH. VOL		
0(PIVH, SAH)	49 (24.50%)	1.0
<30	129 (64.50%)	0.96 (0.49, 1.87) 0.8995
≥30	22 (11.00%)	3.10 (0.91, 10.55) 0.0698
CRP		
≤50	182 (91.92%)	1.0
>50, ≤100	10 (5.05%)	2.74 (0.57, 13.27) 0.2103
>100	6 (3.03%)	3.43 (0.39, 29.93) 0.2655
NLR	13.44 ± 9.43	1.05 (1.02, 1.09) 0.0051
GENDER		
Male	115 (57.50%)	1.0
Female	85 (42.50%)	0.78 (0.44, 1.39) 0.4036
SMOKING		
No	113 (56.50%)	1.0
Yes	87 (43.50%)	1.53 (0.86, 2.74) 0.1503
ALCOHOL		
No	118 (59.00%)	1.0
Yes	82 (41.00%)	1.55 (0.86, 2.79) 0.1432
HTN		
No	47 (23.50%)	1.0
Yes	153 (76.50%)	1.91 (0.99, 3.71) 0.0544
T2DM		
No	169 (84.50%)	1.0
Yes	31 (15.50%)	1.41 (0.63, 3.19) 0.4040
OBSTRUCT. HC		
None (VCR < 0.15)	52 (26.00%)	1.0
Mild (VCR = 0.15–0.23)	41 (20.50%)	2.71 (1.17, 6.31) 0.0204
Severe (VCR > 0.23)	107 (53.50%)	4.67 (2.30, 9.48) < 0.0001
ML. SHIFT		
≤10	170 (85.00%)	1.0
10–15	17 (8.50%)	3.60 (1.00, 12.98) 0.0506
>15	13 (6.50%)	9.25 (1.18, 72.75) 0.0345
Catheter Count		
1	73 (43.98%)	1.0
2	93 (56.02%)	1.09 (0.57, 2.10) 0.7871
RDW	12.92 ± 1.36	1.05 (0.85, 1.30) 0.6416
WBC	12.13 ± 4.75	1.11 (1.04, 1.19) 0.0033
SURG		
No	29 (14.50%)	1.0
Yes	171 (85.50%)	5.25 (2.19, 12.58) 0.0002

NLR, Neutrophil-to-Lymphocyte Ratio; SBP, Systolic Blood Pressure; ICH. VOL, Intracerebral Hemorrhage Volume (PIVH, Punctate Intraventricular Hemorrhage; SAH, Subarachnoid Hemorrhage); CRP, C-Reactive Protein; GENDER, Gender; HTN, Hypertension; T2DM, Type 2 Diabetes Mellitus; OBSTRUCT. HC, Obstructive Hydrocephalus (VCR, Ventricular-Callosal Ratio); ML. SHIFT, Midline Shift; WBC, White Blood Cell Count; RDW, Red Cell Distribution Width; SURG, Surgery.

### 3.3 Association between NLR and prognosis of sIVH

Table 3 reveals the complex associations between catheter duration and infection risk across unadjusted and adjusted statistical models. Neutrophil-to-Lymphocyte Ratio (NLR) emerged as a consistently significant predictor of infection, demonstrating a strong correlation across all models (OR 1.1–1.04, *p* < 0.05). Intracerebral hemorrhage volume (ICH. VOL) showed an increasing infection risk after adjustments, with odds ratios progressively rising from 0.8 to 1.11 (*p* < 0.001). Surgical intervention exhibited a notable association with infection risk in unadjusted and initial adjusted models, but lost statistical significance in the fully adjusted model (OR 5.2 to 2.78, *p* = 0.075). Age and gender displayed minimal impact on infection risk, with marginal variations across different models. These findings underscore the critical role of NLR as a potential biomarker for predicting infection risk in patients with catheter-related complications, highlighting the importance of comprehensive clinical assessments and potential targeted interventions.

**Table 3:** Association between NLR and prognosis of sIVH.

Exposure	Model I (OR, 95%CI, *p*-value)	Model II (OR, 95%CI, *p*-value)	Model III (OR, 95%CI, *p*-value)
Age	1.01 (0.99, 1.04) 0.3037	1.01 (0.98, 1.03) 0.4704	1.03 (1.00, 1.06) 0.0580
Gender			
1	1.0	1.0	1.0
2	0.78 (0.44, 1.39) 0.4036	1.27 (0.55, 2.93) 0.5755	1.24 (0.44, 3.46) 0.6872
NLR			
Q1 (1–8.25)	1.0	1.0	1.0
Q2 (8.43–15.12)	3.71 (1.79, 7.69) 0.0004	4.31 (2.00, 9.26) 0.0002	2.55 (1.03, 6.33) 0.0439
Q3 (15.18–51.88)	3.05 (1.50, 6.20) 0.0020	3.83 (1.79, 8.19) 0.0005	2.39 (0.98, 5.82) 0.0547
ICH. VOL	1.05 (1.02, 1.08) 0.0007	1.05 (1.02, 1.09) 0.0009	1.11 (1.04, 1.19) 0.0016
Surgery			
0	1.0	1.0	1.0
1	5.25 (2.19, 12.58) 0.0002	6.66 (2.65, 16.77) < 0.0001	2.78 (0.90, 8.55) 0.0754

NLR, Neutrophil-to-Lymphocyte Ratio; ICH. VOL, Intracerebral Hemorrhage Volume. Model I: adjust for none. Model II: adjust for: Age; Gender; Smoking; Alcohol; Hypertension; Type 2 Diabetes Mellitus. Model III: adjust for Age; Gender; Smoking; Alcohol; Hypertension; Type 2 Diabetes Mellitus; Intracerebral Hemorrhage Volume; Obstructive Hydrocephalus; Ventricular Hemorrhage; Surgery.

### 3.4 Smooth curve fitting, threshold effect and saturation effect analyses between NLR and prognosis of sIVH

**Figure 2:**
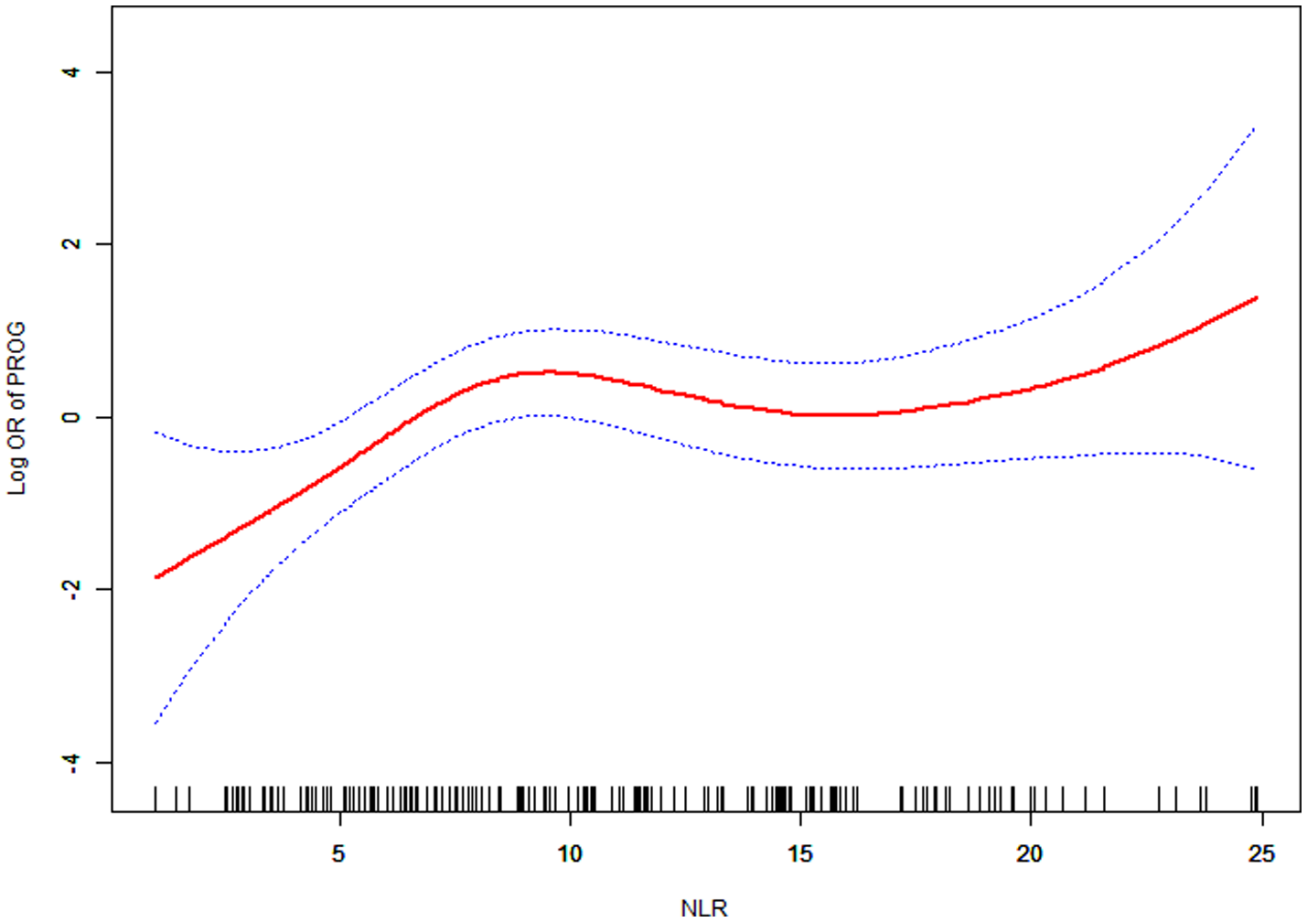
Association between NLR and prognosis of sIVH.

A nonlinear relationship was found between NLR and the risk of poor outcomes in sIVH patients (Figure 2). This was discovered through GAM and smooth curve fitting (adjusted for age, gender, smoking status, alcohol consumption, hypertension, type 2 diabetes mellitus, intracerebral hemorrhage volume, obstructive hydrocephalus, and ventricular hemorrhage volume). To account for the distinct slopes, the data were fitted to a two-piecewise linear regression model. Additionally, we employed a standard linear regression model to fit the data, according to the sensitivity analysis. The log-likelihood ratio test was employed to determine the best-fit model, yielding a *p* value of 0.006, indicating that the two-piecewise linear regression model was more appropriate than the linear model. The inflection point was identified at 8.26 through the implementation of a recursive algorithm. We subsequently calculated effect sizes and confidence intervals for the two segments defined by the inflection point via a two-piecewise linear regression model. Below the inflection point (8.26), each 1-unit increase in NLR was associated with a 49% higher risk of poor outcomes (OR = 1.49, 95% CI: 1.16–1.91, *p* = 0.0018). However, beyond the inflection point at 8.26, NLR had no significant effect on the risk of poor outcomes (OR = 0.95, 95% CI: 0.84–1.07, *p* = 0.4194; Table 4).

**Table 4:** Analysis of threshold effect and saturation effect.

The results of the standard linear and two-piecewise linear regression model prognosis (OR, 95% CI, *p* value)	
Fitting model by standard linear regression	1.09 (1.00, 1. 17) 0.0371
Fitting model by two-piecewise linear regression	8.26
NLR(K) < K> K	1.49 (1.16, 1.91) 0.0018 0.95 (0.84, 1.07) 0.4194
Equation predicted values at break points	1.03 (0.40, 1.66) 0.006
*p-*value for log likelihood ratio test	

Adjust for: Age; Gender; Smoking Status; Alcohol Consumption; Hypertension; Type 2 Diabetes Mellitus; Intracerebral Hemorrhage Volume; Obstructive Hydrocephalus; Ventricular Hemorrhage Volume.

### 3.5 Relationship between between NLR and prognosis of sIVH in different subgroup

**Figure 3:**
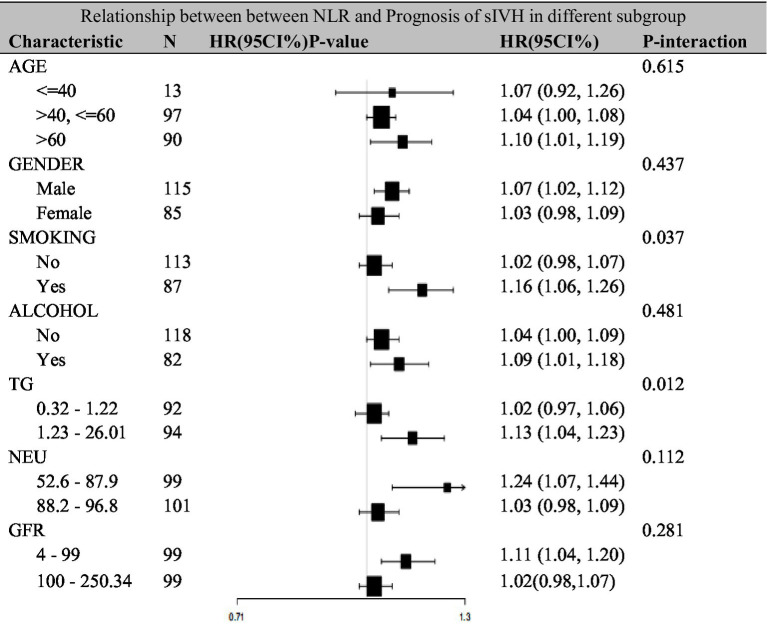
Relationship between between NLR and prognosis of sIVH in different subgroup.

Additionally, a series of sensitivity analyses were conducted to evaluate the stability of the association between NLR and poor outcomes across different subgroups. The study population was stratified by age (≤40, >40 to ≤60, >60 years), gender (male, female), smoking status (yes, no), alcohol consumption (yes, no), TG levels (0.32–1.22, 1.23–26.01), neutrophil percentage (52.6–87.9%, 88.2–96.8%), and GFR (4–99, 100–250.34). In the subgroup analyses, significant interactions were observed for smoking status (P for interaction = 0.037) and TG levels (P for interaction = 0.012), with smokers showing a stronger association (HR = 1.16, 95% CI: 1.06–1.26) compared to non-smokers (HR = 1.02, 95% CI: 0.98–1.07), and patients with higher TG levels (1.23–26.01) demonstrating a stronger association (HR = 1.13, 95% CI: 1.04–1.23) compared to those with lower TG levels (HR = 1.02, 95% CI: 0.97–1.06). Other subgroups, including age (P for interaction = 0.615), gender (P for interaction = 0.437), alcohol consumption (P for interaction = 0.481), neutrophil percentage (P for interaction = 0.112), and GFR (P for interaction = 0.281), did not show significant interaction effects. These findings remained robust after adjusting for potential confounders including demographic characteristics, laboratory parameters, and clinical factors (Figure 3).

## 4 Discussion

This retrospective study represents the first comprehensive investigation of the non-linear relationship between neutrophil-to-lymphocyte ratio (NLR) and prognosis in spontaneous intraventricular hemorrhage (sIVH) patients. Our primary finding reveals a distinctive J-shaped association with a critical inflection point at NLR = 8.26. Below this threshold, each unit increase in NLR was associated with a 49% higher risk of poor outcomes (OR = 1.49, 95% CI: 1.16–1.91, *p* = 0.0018). However, beyond this inflection point, NLR showed no significant additional prognostic value (OR = 0.95, 95% CI: 0.84–1.07, *p* = 0.4194). This threshold effect has important clinical implications for risk stratification in sIVH patients. The identification of 8.26 as a critical cutoff provides clinicians with a concrete reference point for early prognostic assessment and potentially guides therapeutic intervention strategies. Patients with admission NLR values between 1 and 8.26 require careful monitoring as rising NLR correlates with significantly worsening outcomes, while those with NLR > 8.26 may have reached a prognostic “ceiling effect” where additional inflammatory burden does not translate to incrementally worse outcomes.

Lattanzi et al. conducted a study of 346 acute intracerebral hemorrhage patients and found that elevated admission NLR (>4.3) independently predicted 90-day mortality (OR = 2.18, 95% CI: 1.24–3.83), but their analysis assumed a linear relationship without exploring potential threshold effects (23). Zhang et al. reported in a large-scale study of 1,247 ICH patients that each unit increase in NLR was associated with a 12% increased risk of 90-day poor outcomes (OR = 1.12, 95% CI: 1.07–1.18), similarly employing linear modeling approaches (21, 23). Yu et al. found in a prospective study of supratentorial ICH that patients with NLR > 5.0 had significantly worse 3-month functional outcomes (mRS 4–6: 68.2% vs. 41.7%, *p* < 0.001), though this study did not include intraventricular hemorrhage patients (24). More specifically, Shi et al. demonstrated through a meta-analysis that higher NLR values predicted worse clinical outcomes in patients with severe spontaneous basal ganglia hemorrhage, with pooled analysis showing a mortality risk ratio of 2.34 (95% CI: 1.87–2.93) for high versus low NLR groups (25). However, these studies primarily focused on linear relationships, whereas the current study is the first to reveal a non-linear, J-shaped association specifically in patients with spontaneous intraventricular hemorrhage (sIVH). Sun et al. reported in a study of 189 intraventricular hemorrhage patients that admission NLR was an independent predictor of 90-day mortality (HR = 1.15, 95% CI: 1.08–1.23), but this study used continuous variable analysis without identifying specific prognostic thresholds (26).

The J-shaped relationship we observed differs from the predominantly linear associations reported in ischemic stroke literature. Brooks et al. found that admission NLR showed a linear association with 90-day outcomes in ischemic stroke patients undergoing endovascular therapy (OR = 0.89 per unit increase for good outcome) (5). Similarly, Maestrini et al. confirmed a linear relationship between NLR and early neurological deterioration in acute ischemic stroke patients (OR = 1.08 per unit increase) (5, 27). Tokgoz et al. reported that NLR > 2.9 was associated with poor functional outcomes at 3 months in acute ischemic stroke (OR = 3.42, 95% CI: 1.78–6.56), again demonstrating linear predictive patterns (28). This discrepancy suggests that inflammatory response patterns in hemorrhagic versus ischemic stroke may fundamentally differ, with hemorrhagic stroke potentially exhibiting more complex, non-linear inflammatory cascades. Notably, no previous studies have specifically focused on the prognostic value of NLR in sIVH populations, which have unique pathophysiological characteristics including more severe inflammatory burden due to blood–brain barrier disruption and complex ventricular system involvement. Wang et al. studied intraventricular hemorrhage but primarily focused on traumatic cases with a smaller sample size (*n* = 96) and did not perform non-linear analyses (29). This distinction is crucial as sIVH patients often have worse outcomes compared to general ICH populations, potentially due to the direct toxic effects of blood products on ependymal cells and periventricular white matter. Our study is the first to identify a specific prognostic threshold for NLR (8.26) in this special population, providing a more precise tool for clinical decision-making.

The observed J-shaped curve regarding the neutrophil-to-lymphocyte ratio (NLR) can be elucidated through a variety of interconnected pathophysiological mechanisms. In the ascending limb of the curve (NLR 1–8.26), NLR increases due to escalating neutrophil activation and reflects relative lymphopenia. This increase is critical because neutrophils are recognized as the first inflammatory cells to infiltrate brain tissues impacted by hemorrhage, thereby releasing pro-inflammatory cytokines, including TNF-*α*, IL-1β, and IL-6. Additionally, the production of reactive oxygen species and proteolytic enzymes by activated neutrophils exacerbates blood–brain barrier disruption and contributes to secondary brain injury (30, 31). The concurrent lymphopenia observed in these patients indicates systemic immunosuppression following severe brain injury, largely mediated by the activation of the hypothalamic–pituitary–adrenal axis and sympathetic nervous system (31).

Once NLR exceeds the 8.26 threshold, a saturation or ceiling effect may lead to shifts in interpretation regarding inflammatory response management. In this context, the notion of inflammatory saturation becomes salient, suggesting that maximum neutrophil activation has been attained. Consequently, further increases in NLR may not correlate with additional tissue damage but might instead trigger compensatory anti-inflammatory responses, evidenced by the release of anti-inflammatory cytokines such as IL-10 and TGF-*β*, which may counterbalance ongoing inflammatory damage (32). Such dynamics could imply that patients who survive to obtain extremely high NLR values represent a biologically resilient subset or that these values may indicate systemic complications like sepsis or multi-organ failure, where NLR might serve as a broader marker of critical illness rather than being specific to brain inflammation (33).

Our subgroup analyses revealed that smoking status significantly interacts with NLR-outcome associations (*p* = 0.037). Smokers displayed more pronounced NLR-outcome correlations (HR = 1.16) compared to non-smokers (HR = 1.02). This observation aligns with findings suggesting that smoking exacerbates inflammatory responses and deteriorates cerebrovascular outcomes, largely through mechanisms such as endothelial dysfunction and amplification of oxidative stress (34, 35). Additionally, patients with higher triglyceride levels (1.23–26.01 mg/dL) exhibited enhanced NLR-outcome associations (HR = 1.13) compared to their lower triglyceride counterparts (HR = 1.02, P for interaction = 0.012). This indicates a potential synergy between metabolic dysfunction and inflammatory processes, likely exacerbated by enhanced lipid peroxidation and endothelial dysfunction. Hence, NLR’s prognostic value appears to be context-dependent, suggesting greater utility in patients with pre-existing pro-inflammatory conditions (36).

The practical applications stemming from our findings are significant, encompassing key areas of clinical practice. The admission NLR serves as a useful marker for early risk stratification, effectively identifying patients with severe intraventricular hemorrhage (sIVH) who necessitate intensive monitoring. The established threshold of 8.26 provides a framework for therapeutic decision-making related to aggressive interventions, while the J-shaped relationship offers a nuanced perspective for prognostic counseling tailored for families and caregivers (37, 38). Furthermore, utilizing NLR thresholds in future clinical trials could refine patient stratification, ultimately improving study design and the potential for detecting treatment effects (39).

Our study distinguishes itself through various strengths that enhance confidence in the findings presented. It is the first investigation to delve into non-linear relationships of NLR specifically within the sIVH patient population, addressing a critical gap in hemorrhagic stroke literature (40). The analytical rigor applied involved comprehensive statistical adjustment for multiple confounding variables, focusing on clinically relevant values at admission. This approach permits timely clinical applications. Moreover, identifying effect modifiers facilitates personalized interpretations of NLR values, enhancing individual prognostic assessments. Our adequate sample size has been essential in ensuring robust primary analyses and establishing well-defined thresholds (41).

Nonetheless, several limitations must be acknowledged. The retrospective design of a single-center study raises concerns regarding generalizability and possible selection bias, as our patient cohort may diverge from broader sIVH populations, particularly around treatment protocols and baseline characteristics (42). Moreover, we confined our analysis to admission NLR values without accounting for dynamic fluctuations during hospitalization—recognizing that inflammatory markers typically demonstrate rapid evolution following brain injuries. Single time-point measurements inadequately capture the relationship between the inflammatory trajectory and resultant outcomes (43). Despite comprehensive multivariate adjustments, residual confounding remains plausible from unmeasured factors such as genetic variations that could impact inflammatory responses or specific medication effects (44). Although our sample size of 200 patients supports significant primary analyses, it may constrain statistical power for detecting interactions within smaller subgroups, and threshold effect analyses could benefit from validation in larger, multicenter cohorts (45).

Utilizing the 90-day modified Rankin scale as our primary outcome raises additional considerations. Longer-term outcomes and quality of life measures may reveal differing relationships with NLR, potentially presenting a different picture of the clinical implications of high inflammatory states post-sIVH (46). Furthermore, the influence of various extrinsic factors on NLR—including concurrent infections, pharmacological treatments, and individual variations in immune response—may confound interpretations specifically related to brain inflammation (47).

Several intriguing directions for future research arise from this study’s findings. The need for multicenter prospective validation studies examining the 8.26 threshold across diverse patient populations is paramount for affirming generalizability. Additionally, exploring serial NLR measurements and their relationship with clinical outcomes could provide deeper insights into the dynamic nature of inflammatory processes and their prognostic implications (48). Mechanistic investigations into the biological mechanisms governing the J-shaped relationship could significantly enhance our understanding of inflammatory responses in sIVH contexts. Lastly, clinical trials evaluating whether NLR-guided anti-inflammatory interventions improve patient outcomes represent a logical next step to bridge findings with therapeutic advancements. Developing comprehensive prognostic models that incorporate NLR alongside other inflammatory biomarkers could further refine the capacity for accurate risk prediction and bolster clinical decision-making frameworks.

## 5 Conclusion

This study investigated the non-linear relationship between the neutrophil-to-lymphocyte ratio (NLR) and the prognosis of spontaneous intraventricular hemorrhage (sIVH) patients, revealing a J-shaped association. Our findings demonstrate that NLR is an independent prognostic marker for sIVH, with an identified threshold of 8.26. Below this threshold, higher NLR values were significantly associated with an increased risk of poor outcomes, while values above this threshold showed no additional prognostic impact. These results highlight the importance of inflammatory processes in the pathophysiology of sIVH and suggest that NLR could serve as a valuable biomarker for early risk stratification and clinical decision-making.

## Data Availability

The raw data supporting the conclusions of this article will be made available by the authors, without undue reservation.

## Footnotes

1
http://www.R-project.org
